# Distribution characteristics of oral microbiota and its relationship with intestinal microbiota in patients with type 2 diabetes mellitus

**DOI:** 10.3389/fendo.2023.1119201

**Published:** 2023-03-16

**Authors:** Xiao-jing Guo, Shi-xuan Dai, Jin-di Lou, Xu-xiang Ma, Xiao-juan Hu, Li-ping Tu, Ji Cui, Hao Lu, Tao Jiang, Jia-tuo Xu

**Affiliations:** ^1^ School of Basic Medical Sciences, Shanghai University of Traditional Chinese Medicine, Shanghai, China; ^2^ School of Anesthesiology, Naval Medical University, Shanghai, China; ^3^ Shanghai Collaborative Innovation Center of Health Service in Traditional Chinese Medicine, Shanghai University of Traditional Chinese Medicine, Shanghai, China; ^4^ Department of Endocrinology, Shuguang Hospital Affiliated to Shanghai University of Traditional Chinese Medicine, Shanghai, China

**Keywords:** T2DM, oral microbiota, gut microbiota, distribution, relationship (guanxi)

## Abstract

**Introduction:**

Type 2 diabetes mellitus (T2DM) has a high incidence rate globally, increasing the burden of death, disability, and the economy worldwide. Previous studies have found that the compositions of oral and intestinal microbiota changed respectively in T2DM; whether the changes were associated or interacted between the two sites and whether there were some associations between T2DM and the ectopic colonization of oral microbiota in the gut still need to be identified.

**Research design and methods:**

We performed a cross-sectional observational study; 183 diabetes and 74 controls were enrolled. We used high-throughput sequencing technology to detect the V3-V4 region of 16S rRNA in oral and stool samples. The Source Tracker method was used to identify the proportion of the intestinal microbiota that ectopic colonized from the oral cavity.

**Results:**

The oral marker bacteria of T2DM were found, such as *Actinobacteria, Streptococcus, Rothia*, and the intestinal marker bacteria were *Bifidobacterium, Streptococcus*, and *Blautia* at the genus level. Among them, *Actinobacteria* and *Blautia* played a vital role in different symbiotic relationships of oral and intestinal microbiota. The commonly distributed bacteria, such as Firmicutes, Bacteroidetes, and Actinobacteria, were found in both oral and intestine. Moreover, the relative abundance and composition of bacteria were different between the two sites. The glycine betaine degradation I pathway was the significantly up-regulated pathway in the oral and intestinal flora of T2DM. The main serum indexes related to oral and intestinal flora were inflammatory. The relative abundance of Proteobacteria in the intestine and the Spirochete in oral was positively correlated, and the correlation coefficient was the highest, was 0.240 (P<0.01). The proportion of ectopic colonization of oral flora in the gut of T2DM was 2.36%.

**Conclusion:**

The dysbacteriosis exited in the oral and intestine simultaneously, and there were differences and connections in the flora composition at the two sites in T2DM. Ectopic colonization of oral flora in the intestine might relate to T2DM. Further, clarifying the oral-gut-transmitting bacteria can provide an essential reference for diagnosing and treating T2DM in the future.

## 1 Introduction

Diabetes mellitus is a chronic metabolic disorder characterized by impaired insulin secretion, insulin action, or both (1). The number of diabetes patients is increasing, and 5.37 million diabetes patients worldwide in 2021, and about 90% - 95% of the cases were type 2 diabetes mellitus(T2DM) (2, 3). Early diagnosis and intervention of diabetes can delay the disease progression and reduce the incidence and mortality of long-term cardiovascular and cerebrovascular events (4).

The composition of human microorganisms is associated with the physiological and pathological state of the body (5), the oral microbiota dysbiosis is relevant to diabetes (6, 7). At the same time, different sites in the oral cavity have different microbiota compositions (8). Studies have shown that the variation of bacteria in saliva (9), buccal mucosa (10), and dental plaque was associated with T2DM (11). However, there were few studies on the characteristics of tongue-coating microbiota in diabetes. The tongue coating is not only the center of flora interaction between different oral sites (8, 12) but also a potential microbiological bank in the oral cavity (13), and it is convenient and non-invasive to be collected (14). Hence, it may be an excellent site to diagnose and monitor the state of T2DM through the microbiome in the oral cavity.

Oral microbiota is closely related to the gut microbiota, and some oral-gut flora transmitters are in the oral cavity (15, 16). About 10^11^ bacterial cells flow daily from the oral cavity to the stomach. More than 45% of the subject’s oral cavity and feces had a similar microbiota distribution in the Human Microbiome Project (17). The symbiotic flora in the oral cavity and intestine is essential in regulating the immune system (18). In patients with inflammatory bowel disease, the change of the intestinal microbiota can directly or indirectly affect the composition of the oral microbiota by affecting the host immune response (19). In patients with rheumatoid arthritis and osteoarthritis, the microbiota diversity in the oral and intestine decreased simultaneously, and the abundance of *Porphyromonas gingivalis* in the oral cavity and *Prevotella copri* in the gut had the most apparent change (20). Taking *Porphyromonas gingivalis* can induce intestinal flora imbalance and destroy the intestinal epithelial barrier, leading to the invasion of bacteria and bacterial products and aggravating the pathological changes of nonalcoholic fatty liver and gastrointestinal inflammation in mice (21, 22). The distribution of oral-gut microbiota can affect the host’s healthy state. Clarifying the relationships between oral and intestinal flora in patients with T2DM can provide a reference for diagnosing and treating T2DM.

However, oral bacteria had poor colonization ability in the healthy intestine (18). The ectopic colonization of oral bacteria in the intestinal can induce inflammatory-related diseases (23, 24), such as inflammatory bowel disease (25) and colorectal cancer (26). T2DM has the characteristics of chronic low-grade inflammation (27). Studies found that the intestinal permeability increase to bacterial lipopolysaccharide was an important factor triggering the systemic inflammatory response (28). Moreover, short-chain fatty acids produced by intestinal flora play an essential role in intestinal mucosa integrity (29). In T2DM, butyrate production decreased significantly, and intestinal permeability increased (30, 31). Nevertheless, whether the imbalance of intestinal flora is related to the ectopic colonization of oral flora in patients with type 2 diabetes still needs to be studied.

## 2 Study participants

Patients with the age of onset between 18 and 75 years from the endocrinology department of Shuguang Hospital, affiliated with the Shanghai University of Traditional Chinese Medicine, were recruited in 2021. The diagnostic criteria of T2DM conformed to the guideline for the prevention and treatment of T2DM in China (2020 Edition) (4) and the World Health Organization standards FBG≥7mmol/L and/or OGTT≥11.1 mmol/L) (32). We recruited the healthy control group members from the physical examination center of Shuguang Hospital and Shanghai University of Traditional Chinese Medicine in 2021. A healthy state was defined as no acute or chronic diseases, such as hypertension and glycolipid metabolic diseases, no ongoing benign or malignant diseases that may interfere with the study’s objectives, and no oral diseases. The exclusion criteria were as follows: the one who had severe heart, liver, or kidney dysfunction, acute complications of T2DM, malignant tumors, or other internal severe diseases; those who had taken antibiotics or immunosuppressants in the past three months, topical antibiotics used in recent seven days. Oral conditions such as an untreated oral abscess or fungal infection were excluded. The pregnant and lactating patients or the patients with mental illnesses were eliminated in this study.

**Figure 1:**
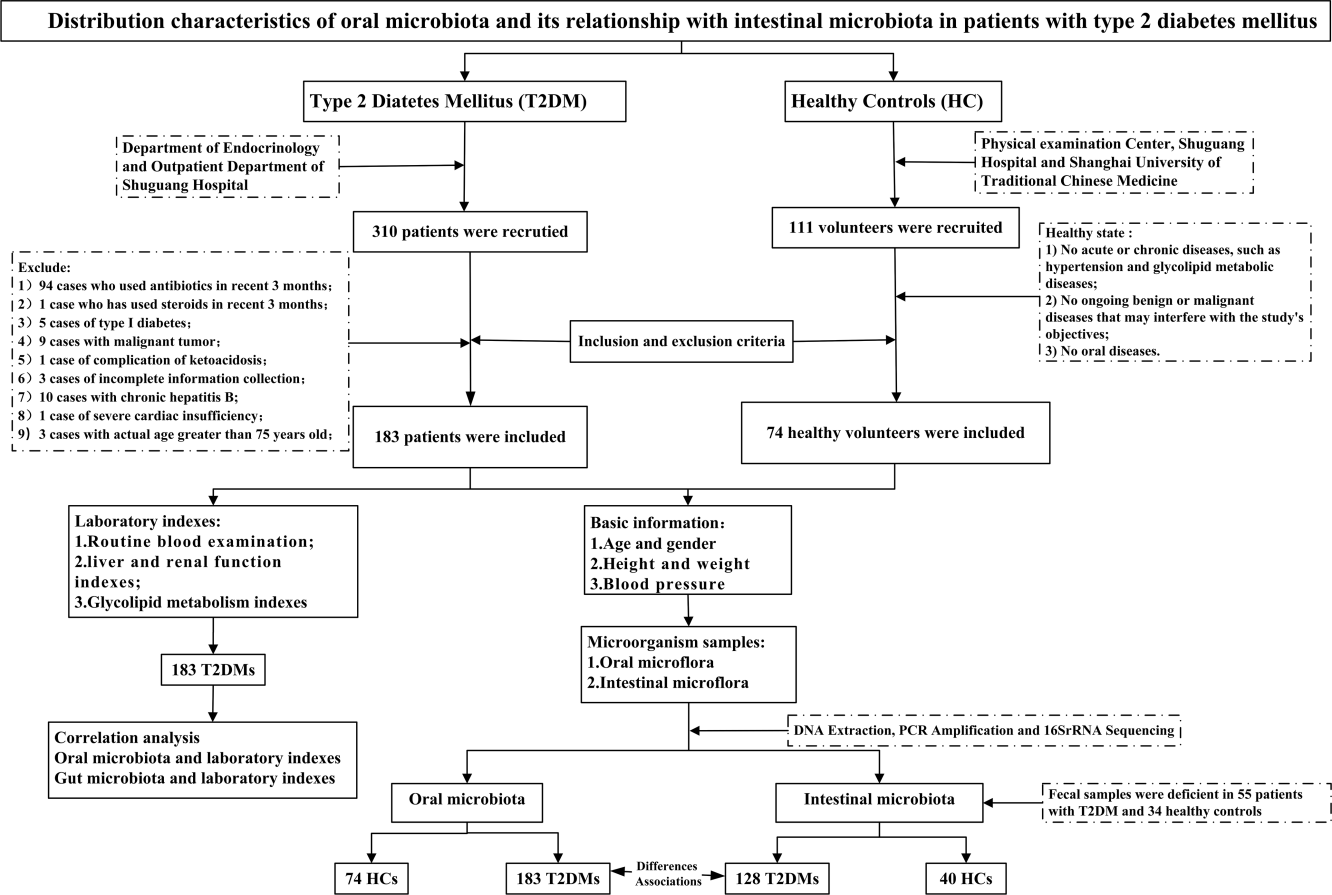
Study design and flow diagram.

183 patients and 74 healthy volunteers participated in this study. 425 microbial samples were collected, including 183 oral and 128 fecal samples from T2DM, 74 oral samples, and 40 fecal samples from the health, **Figure 1**. And a total of 183 blood samples were collected from the T2DM. The basic information about the two groups is in **Supplemental Table 1**. All the participants were requested to sign the informed consent approved by the ethics committee of Shuguang Hospital, Affiliated with Shanghai University of TCM.

## 3 Sample collection

We recorded the participants’ basic information, height, weight, systolic blood pressure, and diastolic blood pressure. We collected the biological blood samples and oral microbiota samples of T2DM from 7:00 am to 9:00 am on an empty stomach. The routine blood examination, liver, and renal function indexes, blood lipids, fasting blood glucose, fasting C-peptide, and glycosylated hemoglobin (HbA1c) by an automatic Beckman Coulter AU5800 biochemical analyzer (Beckman Coulter Inc. Brea, CA, USA), Cobas 8000 e602 module (F. Hoffmann-La Roche, Ltd) and Bole variant II turbo with HPLC method at the Department of Laboratory Medicine, Shuguang Hospital. According to the calculator (HOMA2 Calculator v2.2) provided by the Diabetes Endocrine and Metabolism Research Center of Oxford University, we calculated the insulin resistance index (HOMA2-IR) with fasting blood glucose and fasting C-peptide.

We collected oral microbiota on the middle part of the tongue dorsum with an aseptic pharyngeal swab, rotating back and forth at least ten times. The participants collected the samples in the center of the stool using a fecal sampler in the morning, cryopreserved, and sent them to researchers on the same day. All the pieces were sealed in the sterile and enzyme-free Eppendorf tube on ice and quickly transferred to a minus 80˚C refrigerator within half an hour until sequenced. Anyone who had taken food before oral microbiota collection was asked to wash their mouth with sterile saline at least 2-3 times every 10 seconds, keep abrosia for 2 hours, and then collect.

## 4 DNA extraction, PCR amplification, and sequencing

### 4.1 DNA extraction

According to the manufacturer’s instructions, we extracted total genomic DNA samples with the OMEGA Soil DNA Kit (D5625-01) (Omega Bio-Tek, Norcross, GA, USA). We stored them at minus 20°C before further analysis. The quantity and quality of extracted DNA were measured using a NanoDrop ND-1000 spectrophotometer (Thermo Fisher Scientific, Scientific, Waltham, MA, USA) and agarose gel electrophoresis, respectively.

### 4.2 16S rRNA gene amplicon sequencing

PCR amplification of the bacterial 16S rRNA genes V3-V4 region was performed using forward primer 338F (5’-ACTCCTACGGGAGGCGCAGCA-3’) and the reverse primer 806R(5’-GGACTACHVGGGTWTCTAAT-3’). Sample-specific 7-bp barcodes were integrated into primers for multiple Sequencing. The PCR components include 5μl buffer (5x), 0.25μl Fast pfu DNA Polymerase (5U/μl), 2μl (2.5 mM) dNTPs, 1μl (10 uM) of each Forward and Reverse primer, 1μl DNA template and 14.75μl ddH2O. Thermal cycling included initial denaturation at 98°C for 5 minutes. Then 25 cycles were carried out, including denaturation at 98°C for 30 seconds, annealing at 53°C for 30 seconds and extension at 72°C for 45 seconds, and the final extension at 72°C for 5 minutes. PCR amplicons were purified with Vazyme VAHTSTM DNA Clean Beads (Vazyme, Nanjing, China) and quantified by Quant-iT PicoGreen dsDNA Assay Kit (Invitrogen, Carlsbad, CA, USA). After the individual quantitation, amplicons were pooled in equal amounts. The pair-end 2×250 bp sequencing was performed using the Illumina NovaSeq platform with NovaSeq 6000 SP Reagent Kit (500 cycles) at Shanghai Personal Biotechnology Co., Ltd (Shanghai, China). In addition, the nucleotide sequences of all samples were submitted to the National Center for Biotechnology Information (NCBI) Search database (PRJNA 782768, PRJNA910326).

### 4.3 Sequence analysis

Microbiome bioinformatics was manipulated with QIIME2 2019.4 (33), and there was slight modification according to the official tutorials (https://docs.qiime2.org/2019.4/tutorials/tutorials/). Firstly, we used the demux plugin to demultiplex the raw sequence data, followed by primers cutting with the cutadapt plugin (34). Using the DADA2 plugin, the sequence quality was filtered, denoised, merged, and the chimera removed (35). Non-singleton amplicon sequence variants (ASVs) were aligned with mafft (36) and applied to construct a phylogeny with fasttree2 (37). Alpha diversity metrics [Shannon (38)] and beta diversity metrics [unweighted UniFrac (39)] were estimated using the diversity plugin with samples that were rarefied to 29337 sequences per sample. Taxonomy was assigned to ASVs using the classify-sklearn naive Bayes taxonomy classifier in the feature-classifier plugin (40) against the HOMD Database (41). 34411891 sequences were detected from 425 samples in total, of which the average effective sequence was 72828, the average high-quality sequence was 51016, and the average sequence length was 420. All curves in the sparse curve graph tend to be flat, which indicates that the sequencing depth of all samples in this study is sufficient to reflect the diversity, and further microbiota analysis can be performed (**Supplemental figure 1**).

### 4.4 Bioinformatics and statistical analysis

We utilized a cloud platform (https://www.genescloud.cn/home) for the sequence analyses, including QIIME2 (2019.4), R language (v3.2.0), ggplot2 package, and Python. ASV-level alpha diversity indices and Shannon diversity index was calculated using the ASV table in QIIME2 and visualized as box plots. Kruskal-Wallis rank sum test and Dunn’s test were used as *post-hoc* tests to verify the significance of the difference. ASV-level ranked abundance curves were generated to compare the richness and evenness of ASVs among samples. Beta diversity analysis was performed to investigate the structural variation of microbial communities across models using UniFrac distance metrics (39) and visualized *via* principal coordinate analysis (PCoA) hierarchical clustering (42). The significance of microbiota structure differentiation among groups was assessed by PERMANOVA (Permutational multivariate analysis of variance) (43) using QIIME2. Linear discriminant analysis effect size (LEfSe) was used to detect the differentially abundant taxa among the groups using the default parameters (44). Random forest analysis was applied to discriminate the samples from different groups using QIIME2 with default settings (45, 46). Nested stratified k-fold cross-validation was used for automated hyperparameter optimization and sample prediction. Co-occurrence network analysis was performed by SparCC analysis. The pseudo-count value in SparCC was set to 10^-6^. The cutoff of correlation coefficients was determined as 70 through random matrix theory-based methods as implemented in R package RMThreshold, and network visualization was constructed by Cytoscape (Cytoscape_v3.9.0). The R language was used to analyze the topological structure of the network. The key species were found according to the topological index, and the ZiPi diagram was used for visualization. PICRUSt2 (Phylogenetic Investigation of Communities by Reconstruction of Unobserved States) predicted the microbial function on MetaCyc (https://metacyc.org/).

Furthermore, the difference in the metabolic pathway was analyzed. Microbial traceability was carried out by the method of SourceTracker (47). This method evaluated the distribution of all sequences in source sequences, including unknown source sequences. It used those source sequences to construct the joint distribution of these distributions by the Bayesian algorithm (48). Spearman’s correlation was used to analyze the relationship between the abundance of oral microbiota and intestinal microbiota, microbiota abundance, and biological blood indexes by using IBM SPSS Statistics 26.0. p values < 0.05 were considered significant.

## 5 Research results

### 5.1 Oral *Actinomyces* may exert an essential role in the change of bacteria structure in T2DM

**Figure 2:**
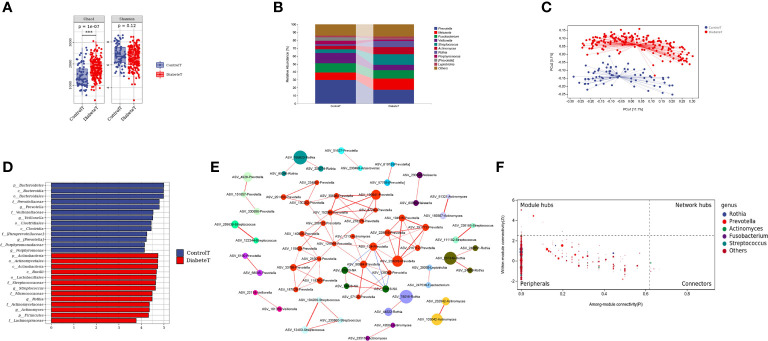
Distribution characteristics of oral microbiota in patients with type 2 diabetes. (Figure **(A)** is the a Diversity results. Figure **(B)** is the composition of oral flora in both groups (top 10 genera). Figure **(C)** shows the PCoA analysis results (each point in the figure represents a sample, and the percentage in the bracket of the coordinate axis represents the proportion of sample difference data that the corresponding coordinate axis can explain). Figure **(D)** is the LEfSe analysis results of oral microbiota in patients with T2DM (the ordinate in the figure was the taxon with significant differences between groups, and the abscissa is the logarithmic score of LDA analysis of each taxon. The taxon is sorted according to the score value. The longer the length is, the more significant the difference between the taxon is. The red bar chart is the diabetes group, the blue bar chart is the control group, and the LDA threshold is 3.5.), Figure **(E)** is the co-abundance network diagram of oral flora in patients with T2DM (Nodes with different colors represent a different module, the larger the nodes, the higher the relative abundance. Red lines represent the positive correlation; blue lines represent the negative correlation, and the thicker the lines, the higher the correlation coefficient). Figure **(F)** is the Zipi diagram of oral flora in patients with T2DM (The X and Y axes, respectively, represent the Pi and Zi values of nodes in the network. The node size is in direct proportion to its abundance. Different colors identify nodes in the top five genera with the highest abundance. The legend on the right shows the bacteria corresponding to each color point.). *** represents P<0.001.

Through the α diversity analysis, we found that oral flora richness in type 2 diabetes patients was higher than that in the control group (P<0.001). There was no significant difference in bacterial uniformity, **Figure 2A**. The oral microbiota was mainly composed of *Prevotella, Neisseria*, and *Fusobacterium* in both groups, **Figure 2B**. And there was a significant difference in the bacteria composition (P<0.001) in **Figure 2C** and **Supplemental Table 2**. Through random forest analysis (ten-fold cross-validation), we found the top 10 bacteria with the most outstanding contribution to the difference between diabetes and control at the genus level, **Supplemental Figure 2**. Among them, the relative abundance of *Streptococcus, Rothia, Cetobacterium*, and *Defluviimonas* in the T2DM group was significantly higher. At the same time, *Prevotella, Proteus, Desulfovibrio*, and *Allobaculum* were markedly lower than that in the control group (P < 0.001), **Supplemental Figure 3**. LEfSe analysis showed that oral *Streptococcus*, *Rothia*, and *Actinomyces* could be used as the marker bacteria in patients with T2DM, **Figure 2D**.

To further clarify the relationships between diverse oral flora in patients with type 2 diabetes, we constructed the co-abundance network (at genus level) of oral microbiota in T2DM using SparCC association network (|r|>0.625, P<0.05), **Figure 2E**. The network was a scale-free network by calculating the topological index, which implied that different oral bacteria in T2DM were mainly linked by the short distance, **Supplemental Figure 4**. The Zi (within-module connectivity) and Pi (among-module connectivity) scores showed that *Prevotella*, *Actinomyces*, and *Fusobacterium* played a vital role in the internal symbiotic networks. *Actinomyces* also was an essential agent among different symbiotic networks, indicating that *Actinomycetes* may play a significant role in the change of microbiota structure in type 2 diabetes, **Figure 2F**.

### 5.2 Intestinal *Blautia* has a close association with T2DM

**Figure 3:**
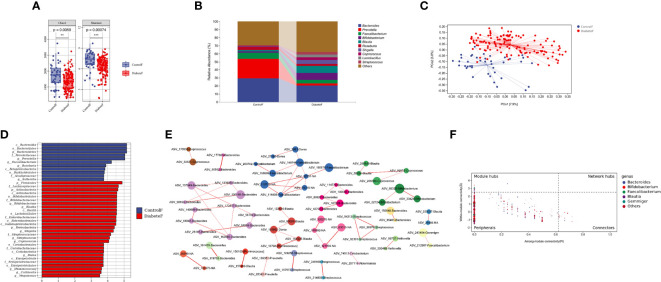
Distribution characteristics of intestinal microbiota in patients with type 2 diabetes (Figure **(A)** is the a Diversity results; Figure **(B)** is the composition of intestinal flora in both groups (top 10 genera); Figure **(C)** is the results of the PeoA analysis; Figure **(D)** is the LEfSe analysis results of intestinal microbiota in patients with T2DM (the LDA threshold is 3.5); Figure **(E)** is the co-abundance network diagram of intestinal flora in patients with T2DM; Figure **(F)** is the Zipi diagram of intestinal flora in patients with T2DM). **represents P<0.01 , *** represents P<0.001.

We compared differences in gut microbiota composition between 128 patients with T2DM and 40 healthy volunteers, **Supplemental Table 3**. The results showed that both uniformity and richness of intestinal microbiota in T2DM patients were significantly lower than in the control group (P<0.001), **Figure 3A**. The intestinal microbiota was mainly composed of *Bacteroides*, *Prevotella*, and *Faecalibacterium* in both groups, **Figure 3B**. Meanwhile, the two groups could clearly distinguish the microbiota composition, and the difference was statistically significant (P=0.001), **Figure 3C** and **Supplemental Table 4**. Through random forest analysis (ten-fold cross-validation), we found the top 10 bacteria with the most significant contribution to the difference between diabetes and control, **Supplemental Figure 5**. Among them, the relative abundance of *Cetobacterium*, *Rothia*, *Shigella*, *Actinomyces*, *Defluvimonas*, and *Blautia* was higher in T2DM, **Supplemental Figure 6**. LEfSe analysis showed that the main intestinal flora in T2DM was Firmicutes, Actinobacteria, Proteobacteria, and Verrucomicrobia at the phylum level, *Bifidobacterium*, *Blautia*, *Shigella*, and *Streptococcus* at the genus level, **Figure 3D**.

Simultaneously, we constructed the co-abundance network of the intestinal microbiota of T2DM, **Figure 3E**. It was a scale-free network, **Supplemental Figure 7**. *Bacteroides*, *Faecalibacterium*, and *Blautia* played essential roles in the inner of various symbiotic networks; *Bacteroidetes* and *Blautia* also played important roles among different symbiotic networks, **Figure 3F**. Based on the above results, *Blautia* was closely related to T2DM and might be essential in changing intestinal microbiota structure in type 2 diabetes.

### 5.3 Differences in the composition of oral and intestinal flora in T2DM

**Figure 4:**
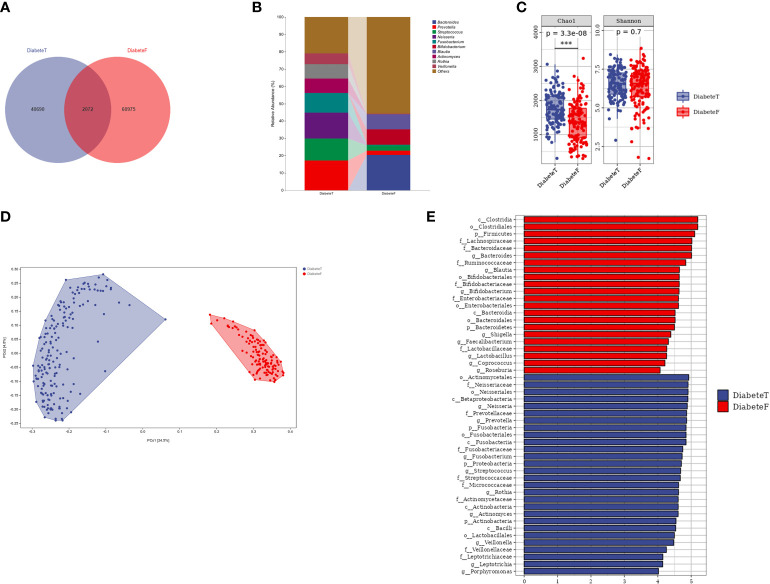
Difference between oral flora and intestinal flora in patients with T2DM. (Figure **(A)** is the Venn diagram of oral and intestinal microbiota at the ASV level. Figure **(B)** is the microbiota in both groups (top 10 genera). Figure **(C)** is the results of a diversity. Figure **(D)** is the result of the PeoA analysis. Figure **(E)** is the LEISe analysis results (the LDA threshold is 4). T represents the tongue-coating microbiota sample, and F represents the intestinal microbiota samples.). *** represents P<0.001.

The oral and gut microbiota compositions were different in patients with T2DM, although some bacteria colonized in both sites simultaneously, such as *Prevotella* and *Streptococcus*, **Figures 4A, B**. Nevertheless, the flora in the two areas differed in the α and β diversity (P<0.001), **Figure 4C, D**, and **Supplemental Table 5**. The random forest results (10-fold cross-verification) showed that *Bacteroides, Leptotrichia, Campylobacter, Ruminococcu*, and *Blautia* were the main bacteria that could distinguish the oral flora from the intestine, **Supplemental Figure 8**. LEfSe analysis showed that *Prevotella, Neisseria, Fusobacterium, Streptococcus*, and *Actinomycetes* were the dominant bacteria in the oral cavity. *Bacteroides, Bifidobacterium, Brautella, Shigella*, and *Clostridium praxis* were the predominant compositions in the intestine (at genus level), **Figure 4E**.

The results of PICRUSt2 showed that Biosynthesis and Degradation were the central metabolic pathways of oral and intestinal microbiota in T2DM, **Supplemental Figures 9**, **10**. The metabolic pathways significantly up-regulated in the oral and gut flora of T2DM were different compared with healthy individuals. However, the glycine betaine degradation I pathway (PWY-3661) was up-regulated in the oral and intestinal flora simultaneously, **Supplemental Figures 11**, **12**.

### 5.4 The intestinal microbiota distribution was associated with oral microbiota in T2DM

The oral and intestinal bacteria with average relative abundance greater than 1% were selected as typical bacteria (ASV level) for correlation analysis in 128 patients with T2DM. A total of 15 ASVs were chosen from the oral, which belonged to *Fusobacterium*, *Prevotella*, *Streptococcus*, *Veillonella*, *Neisseria*, *Rothia*, and *Actinomyces*. 12 ASVs were selected from the intestinal tract: *Shigella*, *Blautia*, *Bifidobacterium*, *Bacteroides*, *Gemmiger*, *Faecalibacterium*, *unclassified_Ruminococcaceae* and *Streptococcus* (at genus level). *Streptococcus* was distributed in the oral and intestinal tract, and the average constituent ratio was 13.4% and 2.93%, respectively.

**Figure 5:**
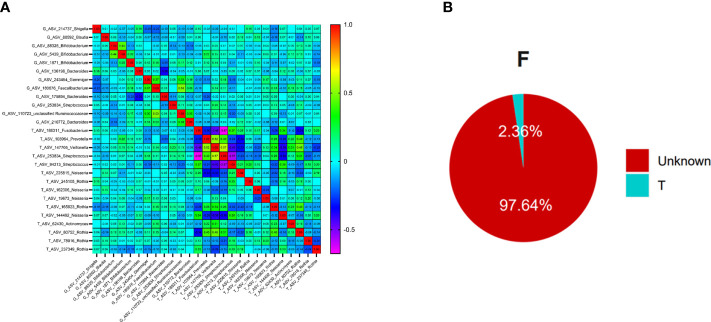
The relationships between oral flora and intestinal flora in T2DM (Figure **(A)** is the correlation beatmap between oral flora and intestinal flora, Figure **(B)** is the results of the Source Tracker, F represents the feces samples, and T represents the tongue-coating samples).

Spearman correlation analysis found that *Prevotella* in the oral was positively correlated with *Bifidobacterium* in the gut, and the correlation coefficient was the highest (r=0.224, P<0.05), **Figure 5A**. At the same time, the top 10 bacteria (at phylum level) co-distributed in both oral and intestinal tract were selected for correlation analysis. The results showed that the correlation coefficient between Spirochaetes in oral and Proteobacteria in the intestinal tract was the highest (r=0.240, P<0.01), **Supplemental Table 6**.

To further explore whether there was ectopic colonization of oral flora in the intestinal tract in patients with T2DM, we used the Source Tracker method to analyze. The results showed that nearly 2.36% of intestinal flora came from the ectopic colonization of tongue-coating flora, which was higher than the results of healthy people (< 0.9%) in the previous study (49), **Figure 5B**.

### 5.5 The relationships between bacterial distributions and blood indices in patients with T2DM

**Figure 6:**
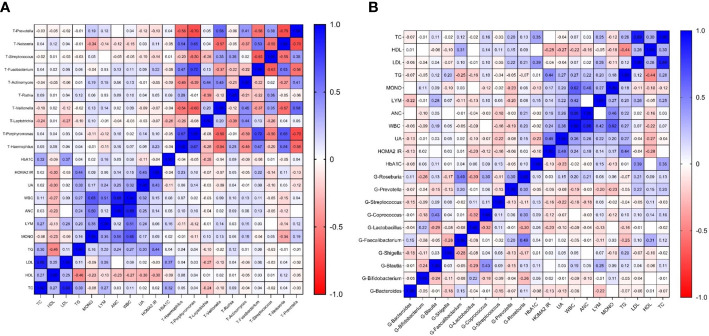
Correlation heatmap of microbiota abundance and serum indexes in T2DM (Figure **(A)** is the correlation heatmap between oral flora and serum indexes; Figure **(B)** is the correlation heatmap between intestinal flora and serum indexes.).

We separately selected the top 10 bacteria (at genus level) in the oral and intestinal tract to analyze their correlation with blood indexes. Among them, *Prevotella* and *Streptococcus* were the typical distribution in both sites. Through correlation analysis, we found that intestinal *Faecalibacterium* had the highest correlation coefficient with HDL (r=0.309 P<0.01). Lymphocytes and white blood cells were the most common indicators related to intestinal flora. *Prevotella* had the most correlations with laboratory indicators and had the highest correlation coefficient with mononuclear cells (r=-0.232, P<0.01), **Figure 6A**. Among the oral microbiota, the correlation coefficient between *Neisseria* and monocytes was the highest (-0.336, P<0.01). The mononuclear cell counts had the most associations with oral microbiota. The bacteria with the most correlated laboratory indicators was *Leptotrichia*, with the highest correlation coefficient with HbA1C (r=-0.275, P<0.01). *Prevotella* distribution in both sites had the exact correlation with monocyte count. Based on these results, we found that the main indexes related to oral and intestinal flora in patients with T2DM were inflammatory, **Figure 6B**.

## 6 Discussion

The microbiota composition is closely related to T2DM. Research shows that even if there is no oral problem, the composition of oral flora and its metabolites in patients with T2DM have also changed compared with healthy people, such as *Porphyromonas gingivalis* and *Prevotella melanogeneica* significantly enriched (50). Using prebiotics to regulate the oral flora of patients with T2DM can recover insulin resistance and glucose intolerance (51). There is a correlation between intestinal microbes and T2DM and its complications (52). Intestinal *Bifidobacterium*, *Bacteroides*, and *Akkermansia* were negatively correlated with T2DM, while *Rumococcus*, *Fusobacterium*, and *Blautia* were positively correlated with T2DM (53). At the same time, oral administration of *Blautia wexlerae* can change the composition of intestinal flora and its metabolic function, reducing obesity and diabetes caused by a high-fat diet (54). These studies indicate that oral and intestinal flora are pathogenic factors of T2DM and potential targets for its treatment.

In this study, we found dysbiosis of oral microbiota in Patients with T2DM. Moreover, the preponderant bacteria distributed in the oral were *Streptococcus, Rothia*, and *Actinomyces* (at genus level), which were consistent with other studies (6, 7, 55). *Streptococcus* is the earliest colonized bacteria in the human body and has a wide distribution throughout the whole body, with a high abundance in the mouth (16, 56, 57). Some oral *Streptococci* can produce acidic substances, which are directly related to the development of dental caries (57). *Streptococci* could increase the possibility of oral diseases under poor blood glucose control, such as periodontitis and dental caries (58, 59). *Actinomyces* was a Gram-positive, obligate anaerobic bacteria and a facultative pathogenic bacteria colonized in the oral cavity, intestine, and skin. It was a common bacteria in the oral cavity (60). In this study, we found that oral actinomyces may be one of the main bacteria-inducing changes in oral flora in patients with T2DM. The pathogenicity of *Actinomyces* may be related to glucose metabolism. It could obtain energy by participating the glycolysis. Moreover, its metabolites could lead to the accumulation of intracellular polysaccharides (61). *Actinomyces* had a close association with gingival and periodontal diseases, which may be an aggravating factor in the onset of diabetes disease (62, 63).

Intestinal dysbacteriosis also occurred in patients with T2DM. *Bifidobacterium, Blautia*, and *Streptococcus* were the predominant bacteria in the intestine. Among them, *Blautia* plays a vital role in the symbiotic relationship of intestinal flora. It is an essential short-chain fatty acid-producing bacteria with an anti-inflammatory effect (64). The high abundance of *Blautia* could increase intestinal permeability, causing an inflammatory response (64). *Blautia* was positively correlated with T2DM (53), which was consistent with the result of this study. However, some studies found that the increase in *Blautia* was closely related to improving glucose and lipid homeostasis in T2DM (65) and could alleviate diabetes and obesity (66, 67). Therefore, further research still needs to clarify the role of *Blautia* in patients with T2DM. *Bifidobacterium* mainly distributes in the intestinal tract of insects and mammals. It was considered beneficial to the health and closely related to the diet habits of the host (68). The use of Acarbose can hinder the hydrolysis and absorption of carbohydrates in the small intestine, which can increase the fermented substrate of microbiota in the distal intestinal, promoting the growth of *Lactobacillus* and *Bifidobacterium* and depleting the original dwelling species, such as *Bacteroides* and *Clostridium* (69). Patients in this study are using or have been treated with related hypoglycemic drugs, influencing the results of this study.

Some bacteria colonized oral and intestinal tracts, such as Firmicutes, Bacteroidetes, and Actinobacteria, consistent with other research results (14, 70). The relative abundance and distribution structure of bacteria were significantly different between the two sites, while they also had some connections and might interact with each other. Spirochaetes and Proteobacteria, distributed in the oral and intestine simultaneously, had a positive correlation. Its correlation coefficient was the highest in the correlation analysis between bacteria co-distributed in both sites (r=0.240, P < 0.01). Spirochetes have a double membrane architecture, and it has the characteristics of both Gram-positive and Gram-negative (71). It contains some pathogenic species, such as Leptospira and Treponema pallidum, closely related to zoonotic leptospirosis and syphilis (72–74). Oral Treponema, the key pathogen related to periodontitis and gingivitis, has more than 60 species (75, 76). Furthermore, periodontitis and gingivitis have a close association with T2DM. The increase of Proteobacteria abundance is closely related to intestinal dysbacteriosis and associated with some inflammatory diseases, such as metabolic disorders and inflammatory bowel disease (77–79). The correlation between oral Spirochete and intestinal Proteobacteria suggests that the pathogenic microorganisms at different human body sites can change synchronously in the state of disease, which provides clues for the monitoring, diagnosing, and treating diseases.

The intestinal native microbiota, mainly probiotics that secrete bacteriocins, antibiotics, and metabolites, can compete for nutrition and space against exogenous pathogens to maintain body health (80). The ectopic colonization of oral flora in the intestine was associated with diseases such as liver cirrhosis (81) and IBD (25). *Prevotella*, co-distributed in the oral cavity and intestine, could colonize in the distal intestine from the oral (82). Oral microflora had the chance to colonize in the intestine, destroying the intestinal epithelial barrier, inducing the excessive secretion of inflammatory cytokines, the destruction of the host immune system, and the induction of immune escape (83). T2DM is characterized by chronic low-grade inflammation (27), closely related to intestinal microflora change and intestinal permeability increase (84). In this study, the proportion of ectopia colonization of oral flora in the intestine reached 2.36%, significantly higher than the healthy (49), indicating that T2DM might correlate with the heterotopic colonization of oral flora in the intestine, which might provide an essential reference for the diagnosis and treatment of type 2 diabetes.

In addition, microbial metabolites are important mediators that can affect the glucose metabolism of the host (85). The results of PICRUSt2 show that the glycine betaine degradation I (PWY-3661) pathway up-regulated significantly in patients with T2DM and synchronized in the oral and intestinal microbiota. This pathway has anti-inflammatory functions in many diseases and has beneficial effects on obesity, diabetes, cancer, and Alzheimer’s disease (86). The plasma glycine was related to insulin resistance; the increased serum glycine could reduce the risk of T2DM. Human glycine is mainly synthesized from glyoxalate, glucose (through serine), and betaine (87). The increased expression of the glycine degradation pathway in the oral and intestinal flora may lead to the decrease of serum glycine levels and promote the occurrence of insulin resistance. Moreover, it indicated that the metabolic function changes of oral and intestinal flora might be similar, and multi-site flora was necessary for further studies.

Although this study revealed the microecological changes of T2DM, it explored the relationships between the oral and intestinal flora. However, there were still some limitations, such as the age span of the patients included was significant, and there were some differences in the distribution of gender and BMI compared to the controls. Meanwhile, we did not explore the heterotopic colonized bacteria directly related to T2DM.

Some studies have found that the structure of microbiota in healthy people will change with age increasing (88, 89). There are differences in microbiota composition between individuals and within individuals. For example, microbial diversity increases with age until the composition of the microbial community is stable at approximately three years of age. Changes in Actinomycetes, Bacteroides, and Firmicutes mainly manifest it. However, the diversity of intestinal flora of the elderly aged over 70 years decreased (90). With age, the α diversity of oral flora shows a descending trend, while the β diversity shows an increasing trend in healthy people (88). It is suggested that age impacts the distribution of flora, especially in diversity. In this study, the average age of T2DM was higher than that of the control group, and α diversity showed that the Chao1 index of oral microbiota was more elevated in T2DM, while the gut microbiota was contrary. Considering the change of flora diversity with age was different between the state of disease and health, the difference in flora composition among other age groups of T2DM needs to be further studied.

Gender had no significant effect on the distribution of oral flora (5, 91) and might affect intestinal flora (92), which was influenced by the grade of obesity (93). With the increase in BMI, the content of intestinal Firmicutes will gradually increase, while the range of Bacteroides will decrease progressively (94). T2DM is one of the significant metabolic complications of obesity, and they share fundamental pathophysiological mechanisms. Controlling obesity can significantly improve glucose metabolism (95). The change in intestinal flora triggers metabolic inflammation in obesity and T2DM (96). These studies suggest that obesity is closely related to T2DM, and the specific changes in their flora still need further hierarchical analysis.

The metagenomic analysis can identify the metabolic pathway of the potential biomarkers. However, using the 16SrRNA methods only to obtain the predicted results of microbial metabolic pathways. Hence, applying the metagenomic analysis in future studies will provide a more helpful basis for diagnosing and treating T2DM with microbiota. Furthermore, we only used correlation analysis to explore the associations between the flora at different sites and the relationship between the flora and blood indicators. The r value needs to be higher, indicating that this method needs to be revised to identify the associations among different indexes. This is also one of the limitations of this study. We will continue to improve the methodology and the study protocol, explore and validate the oral-gut-transmitting microbes in T2DM and study its relevant mechanism in the future.

## 7 Conclusion

In T2DM, dysbacteriosis exited in the oral and intestine simultaneously. Even though the composition of bacteria was different between the two sites, the abundance of bacteria could interact with each other, and the ectopic colonization of oral flora in the intestinal tract was related to T2DM. Further identifying the corresponding oral-gut-transmitting microbes in T2DM may provide some new clues to intervene and may make it possible to evaluate the state of gut microbiota by observing the changes in oral microbiota in T2DM. Meanwhile, controlling the influencing factors, exploring new bioinformatics analysis methods, and conducting animal experiments to clarify the relative microbial mechanism can help prevent and treat T2DM in the future.

## Data availability statement

The datasets presented in this study can be found in online repositories. The names of the repository/repositories and accession number(s) can be found below: https://www.ncbi.nlm.nih.gov/, PRJNA910326 https://www.ncbi.nlm.nih.gov/, PRJNA782768.

## Ethics statement

The studies involving human participants were reviewed and approved by the ethics committee of Shuguang Hospital, Affiliated with Shanghai University of TCM. The patients/participants provided their written informed consent to participate in this study.

## Author contributions

X-JG, S-XD, and J-DL participated in the research design, sample collection, data analysis, and paper writing, contributing equally to this work. X-JH and JC participated in methodological guidance. X-XM and L-PT participated in research design guidance and paper revision. HL, TJ, and J-TX participated in the design and implementation of the research and provided the necessary guidance for data processing, writing, and revision of the paper. All authors contributed to the article and approved the submitted version.

## References

[1] Kerner W Brückel J. Definition, classification and diagnosis of diabetes mellitus. Exp Clin Endocrinol Diabetes (2014) 122(7):384–6. doi: 10.1055/s-0034-1366278 25014088

[2] International diabetes federation atlas (2021). Available at: http://www.diabetesatlas.org.

[3] Ali MK Pearson-Stuttard J Selvin E Gregg EW. Interpreting global trends in type 2 diabetes complications and mortality. Diabetologia (2022) 65(1):3–13. doi: 10.1007/s00125-021-05585-2 34837505 PMC8660730

[4] Society CD. Guideline for the prevention and treatment of type 2 diabetes mellitus in China (2020 edition). Chin J Diabetes Mellitus (2021) 37(04):311–98.

[5] Jiang B Liang X Chen Y Ma T Liu L Li J. Integrating next-generation sequencing and traditional tongue diagnosis to determine tongue coating microbiome. Sci Rep (2012) 2:936. doi: 10.1038/srep00936 23226834 PMC3515809

[6] Ogawa T Honda-Ogawa M Ikebe K Notomi Y Iwamoto Y Shirobayashi I. Characterizations of oral microbiota in elderly nursing home residents with diabetes. J Oral Sci (2017) 59(4):549–55. doi: 10.2334/josnusd.16-0722 28993578

[7] Anbalagan R Srikanth P Mani M Barani R Seshadri KG Janarthanan R. Next generation sequencing of oral microbiota in type 2 diabetes mellitus prior to and after neem stick usage and correlation with serum monocyte chemoattractant-1. Diabetes Res Clin Pract (2017) 130:204–10. doi: 10.1016/j.diabres.2017.06.009 28648853

[8] The Human Microbiome Project Consortium. Structure, function, and diversity of the healthy human microbiome. Nature (2012) 486(7402):207–14. doi: 10.1038/nature11234 PMC3564958 22699609

[9] Goodson JM Hartman ML Shi P Hasturk H Yaskell T Vargas J. The salivary microbiome is altered in the presence of a high salivary glucose concentration. PloS One (2017) 12(3):e0170437. doi: 10.1371/journal.pone.0170437 28249034 PMC5331956

[10] Chen B Wang Z Wang J Su X Yang J Zhang Q. The oral microbiome profile and biomarker in Chinese type 2 diabetes mellitus patients. Endocrine (2020) 68(3):564–72. doi: 10.1007/s12020-020-02269-6 32246318

[11] Matsha TE Prince Y Davids S Chikte U Erasmus RT Kengne AP. Oral microbiome signatures in diabetes mellitus and periodontal disease. J Dental Res (2020) 99(6):658–65. doi: 10.1177/0022034520913818 32298191

[12] Faust K Sathirapongsasuti JF Izard J Segata N Gevers D Raes J. Microbial co-occurrence relationships in the human microbiome. PloS Comput Biol (2012) 8(7):e1002606. doi: 10.1371/journal.pcbi.1002606 22807668 PMC3395616

[13] Tanner AC Milgrom PM Kent R Mokeem SA Page RC Riedy CA. The microbiota of young children from tooth and tongue samples. J Dental Res (2002) 81(1):53–7. doi: 10.1177/002203450208100112 11824414

[14] Li Y Cui J Liu Y Chen K Huang L Liu Y. Oral, tongue-coating microbiota, and metabolic disorders: A novel area of interactive research. Front Cardiovasc Med (2021) 8:730203. doi: 10.3389/fcvm.2021.730203 34490384 PMC8417575

[15] Chen B-Y Lin W-Z Li Y-L Bi C Du L-J Liu Y. Roles of oral microbiota and oral-gut microbial transmission in hypertension. J Adv Res (2022) 43:147–61. doi: 10.1016/j.jare.2022.03.007 PMC9811375 36585105

[16] Zhou Y Gao H Mihindukulasuriya KA La Rosa PS Wylie KM Vishnivetskaya T. Biogeography of the ecosystems of the healthy human body. Genome Biol (2013) 14(1):R1. doi: 10.1186/gb-2013-14-1-r1 23316946 PMC4054670

[17] Segata N Haake SK Mannon P Lemon KP Waldron L Gevers D. Composition of the adult digestive tract bacterial microbiome based on seven mouth surfaces, tonsils, throat and stool samples. Genome Biol (2012) 13(6):R42. doi: 10.1186/gb-2012-13-6-r42 22698087 PMC3446314

[18] Atarashi K Suda W Luo C Kawaguchi T Motoo I Narushima S. Ectopic colonization of oral bacteria in the intestine drives T(H)1 cell induction and inflammation. Sci (New York NY) (2017) 358(6361):359–65. doi: 10.1126/science.aan4526 PMC5682622 29051379

[19] Said HS Suda W Nakagome S Chinen H Oshima K Kim S. Dysbiosis of salivary microbiota in inflammatory bowel disease and its association with oral immunological biomarkers. DNA Res Int J Rapid Publ Rep Genes Genomes (2014) 21(1):15–25. doi: 10.1093/dnares/dst037 PMC3925391 24013298

[20] Lorenzo D GianVincenzo Z Carlo Luca R Karan G Jorge V Roberto M. Oral-gut microbiota and arthritis: Is there an evidence-based axis? J Clin Med (2019) 8(10). doi: 10.3390/jcm8101753 PMC6832398 31652577

[21] Yamazaki K Kato T Tsuboi Y Miyauchi E Suda W Sato K. Oral pathobiont-induced changes in gut microbiota aggravate the pathology of nonalcoholic fatty liver disease in mice. Front Immunol (2021) 12:766170. doi: 10.3389/fimmu.2021.766170 34707622 PMC8543001

[22] Tsuzuno T Takahashi N Yamada-Hara M Yokoji-Takeuchi M Sulijaya B Aoki-Nonaka Y. Ingestion of porphyromonas gingivalis exacerbates colitis *via* intestinal epithelial barrier disruption in mice. J Periodontal Res (2021) 56(2):275–88. doi: 10.1111/jre.12816 33512709

[23] Cao X. Intestinal inflammation induced by oral bacteria. Sci (New York NY) (2017) 358(6361):308–9. doi: 10.1126/science.aap9298 29051367

[24] Kato T Yamazaki K Nakajima M Date Y Kikuchi J Hase K. Oral administration of porphyromonas gingivalis alters the gut microbiome and serum metabolome. mSphere (2018) 3(5):e00460-18. doi: 10.1128/mSphere.00460-18 30333180 PMC6193602

[25] Singhal S Dian D Keshavarzian A Fogg L Fields JZ Farhadi A. The role of oral hygiene in inflammatory bowel disease. Dig Dis Sci (2011) 56(1):170–5. doi: 10.1007/s10620-010-1263-9 20458622

[26] Koliarakis I Messaritakis I Nikolouzakis TK Hamilos G Souglakos J Tsiaoussis J. Oral bacteria and intestinal dysbiosis in colorectal cancer. Int J Mol Sci (2019) 20(17):4146. doi: 10.3390/ijms20174146 31450675 PMC6747549

[27] Hotamisligil GS. Inflammation and metabolic disorders. Nature (2006) 444(7121):860–7. doi: 10.1038/nature05485 17167474

[28] Minihane AM Vinoy S Russell WR Baka A Roche HM Tuohy KM. Low-grade inflammation, diet composition and health: Current research evidence and its translation. Br J Nutr (2015) 114(7):999–1012. doi: 10.1017/s0007114515002093 26228057 PMC4579563

[29] Blaak EE Canfora EE Theis S Frost G Groen AK Mithieux G. Short chain fatty acids in human gut and metabolic health. Benef Microbes (2020) 11(5):411–55. doi: 10.3920/bm2020.0057 32865024

[30] Horton F Wright J Smith L Hinton PJ Robertson MD. Increased intestinal permeability to oral chromium (51 cr) -EDTA in human type 2 diabetes. Diabetic Med J Br Diabetic Assoc (2014) 31(5):559–63. doi: 10.1111/dme.12360 24236770

[31] Snelson M de Pasquale C Ekinci EI Coughlan MT. Gut microbiome, prebiotics, intestinal permeability and diabetes complications. Best Pract Res Clin Endocrinol Metab (2021) 35(3):101507. doi: 10.1016/j.beem.2021.101507 33642218

[32] Alberti KG Zimmet PZ. Definition, diagnosis and classification of diabetes mellitus and its complications. part 1: Diagnosis and classification of diabetes mellitus provisional report of a WHO consultation. Diabetic Med J Br Diabetic Assoc (1998) 15(7):539–53. doi: 10.1002/(sici)1096-9136(199807)15:7<539::Aid-dia668>3.0.Co;2-s 9686693

[33] Bolyen E Rideout JR Dillon MR Bokulich NA Abnet CC Al-Ghalith GA. Reproducible, interactive, scalable and extensible microbiome data science using QIIME 2. Nat Biotechnol (2019) 37(8):852–7. doi: 10.1038/s41587-019-0209-9 PMC7015180 31341288

[34] Martin M. CUTADAPT removes adapter sequences from high-throughput sequencing reads. EMBnetjournal (2011) 17:10–2. doi: 10.14806/ej.17.1.200

[35] Callahan BJ McMurdie PJ Rosen MJ Han AW Johnson AJ Holmes SP. DADA2: High-resolution sample inference from illumina amplicon data. Nat Methods (2016) 13(7):581–3. doi: 10.1038/nmeth.3869 PMC4927377 27214047

[36] Katoh K Misawa K Kuma K Miyata T. MAFFT: a novel method for rapid multiple sequence alignment based on fast Fourier transform. Nucleic Acids Res (2002) 30(14):3059–66. doi: 10.1093/nar/gkf436 PMC135756 12136088

[37] Price MN Dehal PS Arkin AP. FastTree: Computing large minimum evolution trees with profiles instead of a distance matrix. Mol Biol Evol (2009) 26(7):1641–50. doi: 10.1093/molbev/msp077 PMC2693737 19377059

[38] Shannon CE. A mathematical theory of communication. Bell System Tech J (1948) 27(3):379–423. doi: 10.1002/j.1538-7305.1948.tb01338.x

[39] Lozupone C Knight R. UniFrac: A new phylogenetic method for comparing microbial communities. Appl Environ Microbiol (2005) 71(12):8228–35. doi: 10.1128/aem.71.12.8228-8235.2005 PMC1317376 16332807

[40] Bokulich NA Kaehler BD Rideout JR Dillon M Bolyen E Knight R. Optimizing taxonomic classification of marker-gene amplicon sequences with QIIME 2's q2-feature-classifier plugin. Microbiome (2018) 6(1):90. doi: 10.1186/s40168-018-0470-z 29773078 PMC5956843

[41] FE I Huang Y Chen T Lin M Kokaras A Dewhirst FE. Construction of habitat-specific training sets to achieve species-level assignment in 16S rRNA gene datasets. Microbiome (2020) 8(1):65. doi: 10.1186/s40168-020-00841-w 32414415 PMC7291764

[42] Ramette A. Multivariate analyses in microbial ecology. FEMS Microbiol Ecol (2007) 62(2):142–60. doi: 10.1111/j.1574-6941.2007.00375.x PMC2121141 17892477

[43] McArdle BH Anderson MJ. Fitting multivariate models to community data: A comment on distance&acirc;based redundancy analysis. Ecology (2001) 82(1):290–7. doi: 10.1890/0012-9658(2001)082[0290:FMMTCD]2.0.CO;2

[44] Segata N Izard J Waldron L Gevers D Miropolsky L Garrett WS. Metagenomic biomarker discovery and explanation. Genome Biol (2011) 12(6):R60. doi: 10.1186/gb-2011-12-6-r60 21702898 PMC3218848

[45] Breiman L. Random forests. Mach Learn (2001) 45(1):5–32. doi: 10.1023/A:1010933404324

[46] Liaw A Wiener M. Classification and regression by RandomForest. Forest (2001) 23:18–22.

[47] Knights D Kuczynski J Charlson ES Zaneveld J Mozer MC Collman RG. Bayesian Community-wide culture-independent microbial source tracking. Nat Methods (2011) 8(9):761–3. doi: 10.1038/nmeth.1650 PMC3791591 21765408

[48] McGhee JJ Rawson N Bailey BA Fernandez-Guerra A Sisk-Hackworth L Kelley ST. Meta-SourceTracker: Application of Bayesian source tracking to shotgun metagenomics. PeerJ (2020) 8:e8783. doi: 10.7717/peerj.8783 32231882 PMC7100590

[49] Guo X-J Jiang T Ma X-x Hu X-j Huang J-b Cui L-t. Relationships between diurnal changes of tongue coating microbiota and intestinal microbiota. Front Cell Infect Microbiol (2022) 12. doi: 10.3389/fcimb.2022.813790 PMC9008461 35433494

[50] Li Y Qian F Cheng X Wang D Wang Y Pan Y. Dysbiosis of oral microbiota and metabolite profiles associated with type 2 diabetes mellitus. Microbiol Spectr (2023) 11(1):e0379622. doi: 10.1128/spectrum.03796-22 36625596 PMC9927158

[51] Bahadoran Z Mirmiran P Carlström M Ghasemi A. Inorganic nitrate: A potential prebiotic for oral microbiota dysbiosis associated with type 2 diabetes. Nitric Oxide Biol Chem (2021) 116:38–46. doi: 10.1016/j.niox.2021.09.001 34506950

[52] Iatcu CO Steen A Covasa M. Gut microbiota and complications of type-2 diabetes. Nutrients (2021) 14(1):166. doi: 10.3390/nu14010166 35011044 PMC8747253

[53] Gurung M Li Z You H Rodrigues R Jump DB Morgun A. Role of gut microbiota in type 2 diabetes pathophysiology. EBioMedicine (2020) 51:102590. doi: 10.1016/j.ebiom.2019.11.051 31901868 PMC6948163

[54] Hosomi K Saito M Park J Murakami H Shibata N Ando M. Oral administration of blautia wexlerae ameliorates obesity and type 2 diabetes *via* metabolic remodeling of the gut microbiota. Nat Commun (2022) 13(1):4477. doi: 10.1038/s41467-022-32015-7 35982037 PMC9388534

[55] Sheng L. Metagenomic analysis of yellow thick and greasy tongue coating in patients with type 2 diabetes and healthy people [D]. Beijing Univ Chin Med (2020) 2.

[56] Gao L Xu T Huang G Jiang S Gu Y Chen F. Oral microbiomes: more and more importance in oral cavity and whole body. Protein Cell (2018) 9(5):488–500. doi: 10.1007/s13238-018-0548-1 29736705 PMC5960472

[57] Abranches J Zeng L Kajfasz JK Palmer SR Chakraborty B Wen ZT. Biology of oral streptococci. Microbiol Spectr (2018) 6(5):10.1128/microbiolspec.GPP3-0042-2018. doi: 10.1128/microbiolspec.GPP3-0042-2018 PMC6287261 30338752

[58] Syrjälä AM Niskanen MC Ylöstalo P Knuuttila ML. Metabolic control as a modifier of the association between salivary factors and dental caries among diabetic patients. Caries Res (2003) 37(2):142–7. doi: 10.1159/000069020 12652052

[59] Nabee Z Jeewon R Pugo-Gunsam P. Oral dysbacteriosis in type 2 diabetes and its role in the progression to cardiovascular disease. Afr Health Sci (2017) 17(4):1082–91. doi: 10.4314/ahs.v17i4.16 PMC5870297 29937879

[60] Sharma N Bhatia S Sodhi AS Batra N. Oral microbiome and health. AIMS Microbiol (2018) 4(1):42–66. doi: 10.3934/microbiol.2018.1.42 31294203 PMC6605021

[61] Li J Li Y Zhou Y Wang C Wu B Wan J. Actinomyces and alimentary tract diseases: A review of its biological functions and pathology. BioMed Res Int (2018) 2018:3820215. doi: 10.1155/2018/3820215 30225251 PMC6129341

[62] Bronzato JD Bomfim RA Hayasida GZP Cúri M Estrela C Paster BJ. Analysis of microorganisms in periapical lesions: A systematic review and meta-analysis. Arch Oral Biol (2021) 124:105055. doi: 10.1016/j.archoralbio.2021.105055 33588190

[63] Minty M Canceil T Serino M Burcelin R Tercé F Blasco-Baque V. Oral microbiota-induced periodontitis: A new risk factor of metabolic diseases. Rev Endocr Metab Disord (2019) 20(4):449–59. doi: 10.1007/s11154-019-09526-8 31741266

[64] Zhao T Zhan L Zhou W Chen W Luo J Zhang L. The effects of erchen decoction on gut microbiota and lipid metabolism disorders in zucker diabetic fatty rats. Front Pharmacol (2021) 12:647529. doi: 10.3389/fphar.2021.647529 34366839 PMC8339961

[65] Tong X Xu J Lian F Yu X Zhao Y Xu L. Structural alteration of gut microbiota during the amelioration of human type 2 diabetes with hyperlipidemia by metformin and a traditional Chinese herbal formula: A multicenter, randomized, open label clinical trial. mBio (2018) 9(3):e02392-17. doi: 10.1128/mBio.02392-17 29789365 PMC5964358

[66] Liu X Mao B Gu J Wu J Cui S Wang G. Blautia-a new functional genus with potential probiotic properties? Gut Microbes (2021) 13(1):1–21. doi: 10.1080/19490976.2021.1875796 PMC7872077 33525961

[67] Rodriguez J Hiel S Neyrinck AM Le Roy T Pötgens SA Leyrolle Q. Discovery of the gut microbial signature driving the efficacy of prebiotic intervention in obese patients. Gut (2020) 69(11):1975–87. doi: 10.1136/gutjnl-2019-319726 PMC7569399 32041744

[68] Satti M Modesto M Endo A Kawashima T Mattarelli P Arita M. Host-diet effect on the metabolism of bifidobacterium. Genes (Basel) (2021) 12(4):609. doi: 10.3390/genes12040609 33924280 PMC8074910

[69] Gu Y Wang X Li J Zhang Y Zhong H Liu R. Analyses of gut microbiota and plasma bile acids enable stratification of patients for antidiabetic treatment. Nat Commun (2017) 8(1):1785. doi: 10.1038/s41467-017-01682-2 29176714 PMC5702614

[70] Jandhyala SM Talukdar R Subramanyam C Vuyyuru H Sasikala M Nageshwar Reddy D. Role of the normal gut microbiota. World J Gastroenterol (2015) 21(29):8787–803. doi: 10.3748/wjg.v21.i29.8787 PMC4528021 26269668

[71] Haake DA. Spirochaetal lipoproteins and pathogenesis. Microbiol (Reading) (2000) 146(Pt 7):1491–504. doi: 10.1099/00221287-146-7-1491 PMC2664406 10878114

[72] Nakamura S. Spirochete flagella and motility. Biomolecules. (2020) 10(4):550. doi: 10.3390/biom10040550. 32260454 PMC7225975

[73] Cagliero J Villanueva S Matsui M. Leptospirosis pathophysiology: Into the storm of cytokines. Front Cell Infect Microbiol (2018) 8:204. doi: 10.3389/fcimb.2018.00204 29974037 PMC6019470

[74] Coburn J Picardeau M Woods CW Veldman T Haake DA. Pathogenesis insights from an ancient and ubiquitous spirochete. PloS Pathog (2021) 17(10):e1009836. doi: 10.1371/journal.ppat.1009836 34673833 PMC8530280

[75] Yousefi L Leylabadlo HE Pourlak T Eslami H Taghizadeh S Ganbarov K. Oral spirochetes: Pathogenic mechanisms in periodontal disease. Microbial Pathogen (2020) 144:104193. doi: 10.1016/j.micpath.2020.104193 32304795

[76] Kurniyati K Li C. Genetic manipulations of oral spirochete treponema denticola. Methods Mol Biol (Clifton NJ) (2021) 2210:15–23. doi: 10.1007/978-1-0716-0939-2_2 PMC7925132 32815123

[77] Rizzatti G Lopetuso LR Gibiino G Binda C Gasbarrini A. Proteobacteria: A common factor in human diseases. BioMed Res Int (2017) 2017:9351507. doi: 10.1155/2017/9351507 29230419 PMC5688358

[78] Shin NR Whon TW Bae JW. Proteobacteria: microbial signature of dysbiosis in gut microbiota. Trends Biotechnol (2015) 33(9):496–503. doi: 10.1016/j.tibtech.2015.06.011 26210164

[79] Sun D Ge X Tang S Liu Y Sun J Zhou Y. Bacterial characteristics of intestinal tissues from patients with crohn's disease. Front Cell Infect Microbiol (2021) 11:711680. doi: 10.3389/fcimb.2021.711680 34869050 PMC8635149

[80] Rangan KJ Hang HC. Biochemical mechanisms of pathogen restriction by intestinal bacteria. Trends Biochem Sci (2017) 42(11):887–98. doi: 10.1016/j.tibs.2017.08.005 PMC6038137 28927699

[81] Acharya C Sahingur SE Bajaj JS. Microbiota, cirrhosis, and the emerging oral-gut-liver axis. JCI Insight (2017) 2(19):e94416. doi: 10.1172/jci.insight.94416 28978799 PMC5841881

[82] Tett A Pasolli E Masetti G Ercolini D Segata N. Prevotella diversity, niches and interactions with the human host. Nat Rev Microbiol (2021) 19(9):585–99. doi: 10.1038/s41579-021-00559-y PMC11290707 34050328

[83] Qi Y Wu HM Yang Z Zhou YF Jin L Yang MF. New insights into the role of oral microbiota dysbiosis in the pathogenesis of inflammatory bowel disease. Dig Dis Sci (2022) 67(1):42–55. doi: 10.1007/s10620-021-06837-2 33527328

[84] Cani PD Bibiloni R Knauf C Waget A Neyrinck AM Delzenne NM. Changes in gut microbiota control metabolic endotoxemia-induced inflammation in high-fat diet-induced obesity and diabetes in mice. Diabetes (2008) 57(6):1470–81. doi: 10.2337/db07-1403 18305141

[85] Zhu T Goodarzi MO. Metabolites linking the gut microbiome with risk for type 2 diabetes. Curr Nutr Rep (2020) 9(2):83–93. doi: 10.1007/s13668-020-00307-3 PMC7282969 32157661

[86] Zhao G He F Wu C Li P Li N Deng J. Betaine in inflammation: Mechanistic aspects and applications. Front Immunol (2018) 9:1070. doi: 10.3389/fimmu.2018.01070 29881379 PMC5976740

[87] Adeva-Andany M Souto-Adeva G Ameneiros-Rodríguez E Fernández-Fernández C Donapetry-García C Domínguez-Montero A. Insulin resistance and glycine metabolism in humans. Amino Acids (2018) 50(1):11–27. doi: 10.1007/s00726-017-2508-0 29094215

[88] Liu S Wang Y Zhao L Sun X Feng Q. Microbiome succession with increasing age in three oral sites. Aging (Albany NY) (2020) 12(9):7874–907. doi: 10.18632/aging.103108 PMC7244077 32379704

[89] Kim S Jazwinski SM. The gut microbiota and healthy aging: A mini-review. Gerontology (2018) 64(6):513–20. doi: 10.1159/000490615 PMC6191326 30025401

[90] Rinninella E Raoul P Cintoni M Franceschi F Miggiano GAD Gasbarrini A. What is the healthy gut microbiota composition? a changing ecosystem across age, environment, diet, and diseases. Microorganisms (2019) 7(1):14. doi: 10.3390/microorganisms7010014 30634578 PMC6351938

[91] Minty M Loubières P Canceill T Azalbert V Burcelin R Tercé F. Gender-associated differences in oral microbiota and salivary biochemical parameters in response to feeding. J Physiol Biochem (2021) 77(1):155–66. doi: 10.1007/s13105-020-00757-x 32648199

[92] Kushak RI Winter HS. Gut microbiota and gender in autism spectrum disorders. Curr Pediatr Rev (2020) 16(4):249–54. doi: 10.2174/1573396316999200727123026 32720604

[93] Haro C Rangel-Zúñiga OA Alcalá-Díaz JF Gómez-Delgado F Pérez-Martínez P Delgado-Lista J. Intestinal microbiota is influenced by gender and body mass index. PloS One (2016) 11(5):e0154090. doi: 10.1371/journal.pone.0154090 27228093 PMC4881937

[94] Koliada A Syzenko G Moseiko V Budovska L Puchkov K Perederiy V. Association between body mass index and Firmicutes/Bacteroidetes ratio in an adult Ukrainian population. BMC Microbiol (2017) 17(1):120. doi: 10.1186/s12866-017-1027-1 28532414 PMC5440985

[95] Lingvay I Sumithran P Cohen RV le Roux CW. Obesity management as a primary treatment goal for type 2 diabetes: Time to reframe the conversation. Lancet (2022) 399(10322):394–405. doi: 10.1016/s0140-6736(21)01919-x 34600604

[96] Scheithauer TPM Rampanelli E Nieuwdorp M Vallance BA Verchere CB van Raalte DH. Gut microbiota as a trigger for metabolic inflammation in obesity and type 2 diabetes. Front Immunol (2020) 11:571731. doi: 10.3389/fimmu.2020.571731 33178196 PMC7596417

