# Supplementary material for: Distribution characteristics of oral microbiota and its relationship with intestinal microbiota in patients with type 2 diabetes mellitus
Source: Front Endocrinol (Lausanne). 2023 Mar 16;14:1119201. doi: 10.3389/fendo.2023.1119201 (PMC10072265; doi:10.3389/fendo.2023.1119201)
Supplement: Supplementary file 1 [file DataSheet_1.docx]

**Distribution characteristics and relationships between oral and intestinal microbiota in patients with type 2 diabetes mellitus**

Supplementary Material

# Supplementary Figures and Tables

## Supplementary Figures


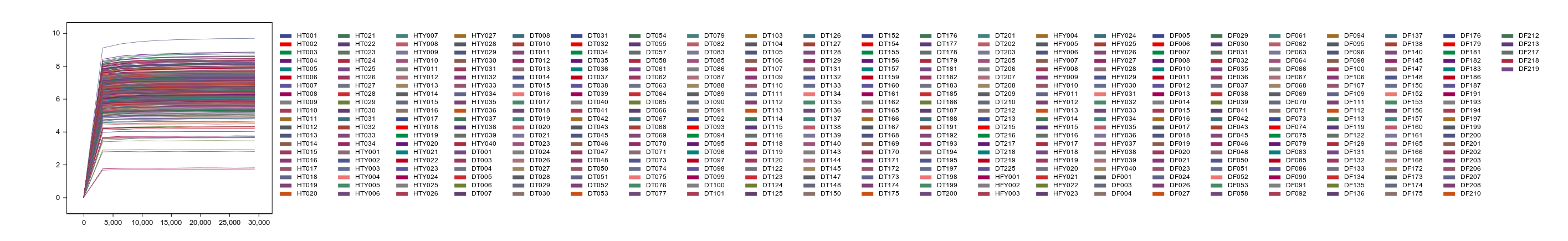


**Supplementary Figure 1.** **The rarefaction curve** （The abscissa is the leveling depth, and the ordinate is the Shannon index. Lines with different colors represent different samples.）


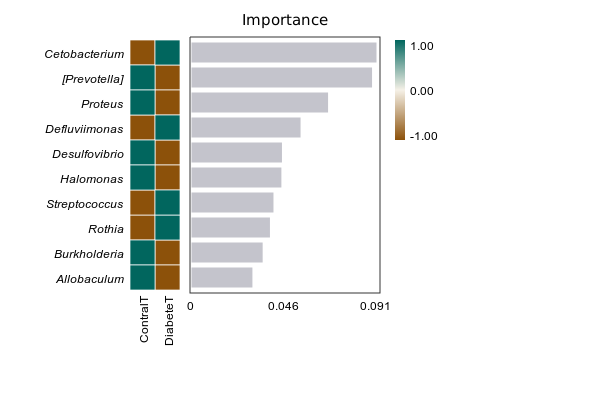


**Supplementary Figure 2. Random forest graph of oral microbiota** (The abscissa of the histogram is the score of the importance of species to the classifier model, and the ordinate is the name of different genera; The heat map shows the abundance of genus in each subgroup, T represents the tongue coating samples.)

**Supplementary Figure 3. The difference of oral microbiota abundance between two groups**

(***means *P* < 0.001)


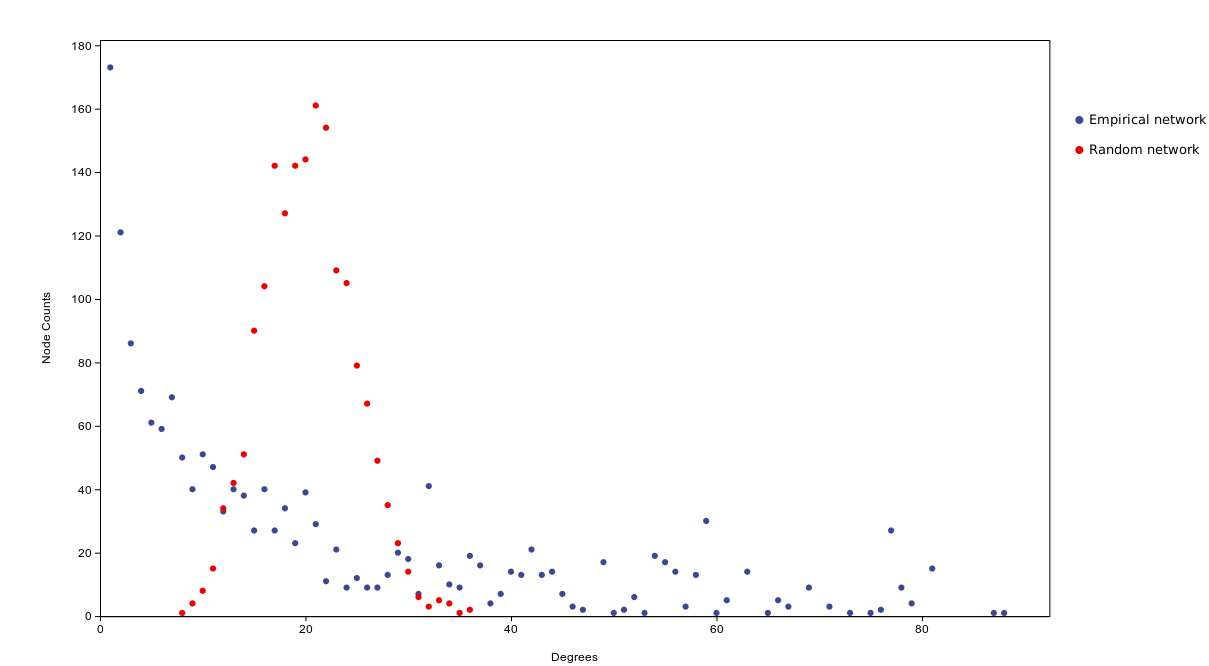


**Supplementary Figure 4. Degree distribution of oral flora in patients with type 2 diabetes（**The abscissa is the degree of the node, and the ordinate is the number of nodes with corresponding degrees.）

**
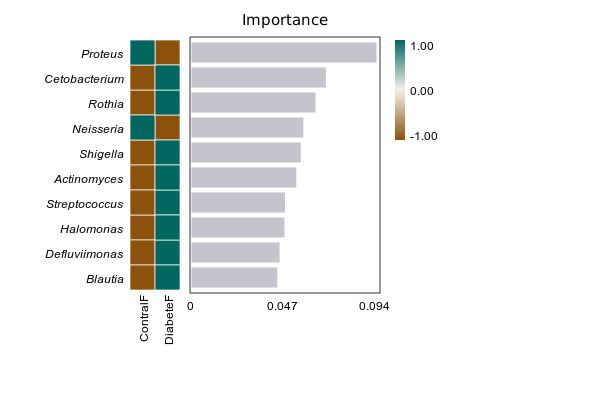
**

**Supplementary Figure 5. Random forest graph of intestinal microbiota** (F represents the fecal samples).


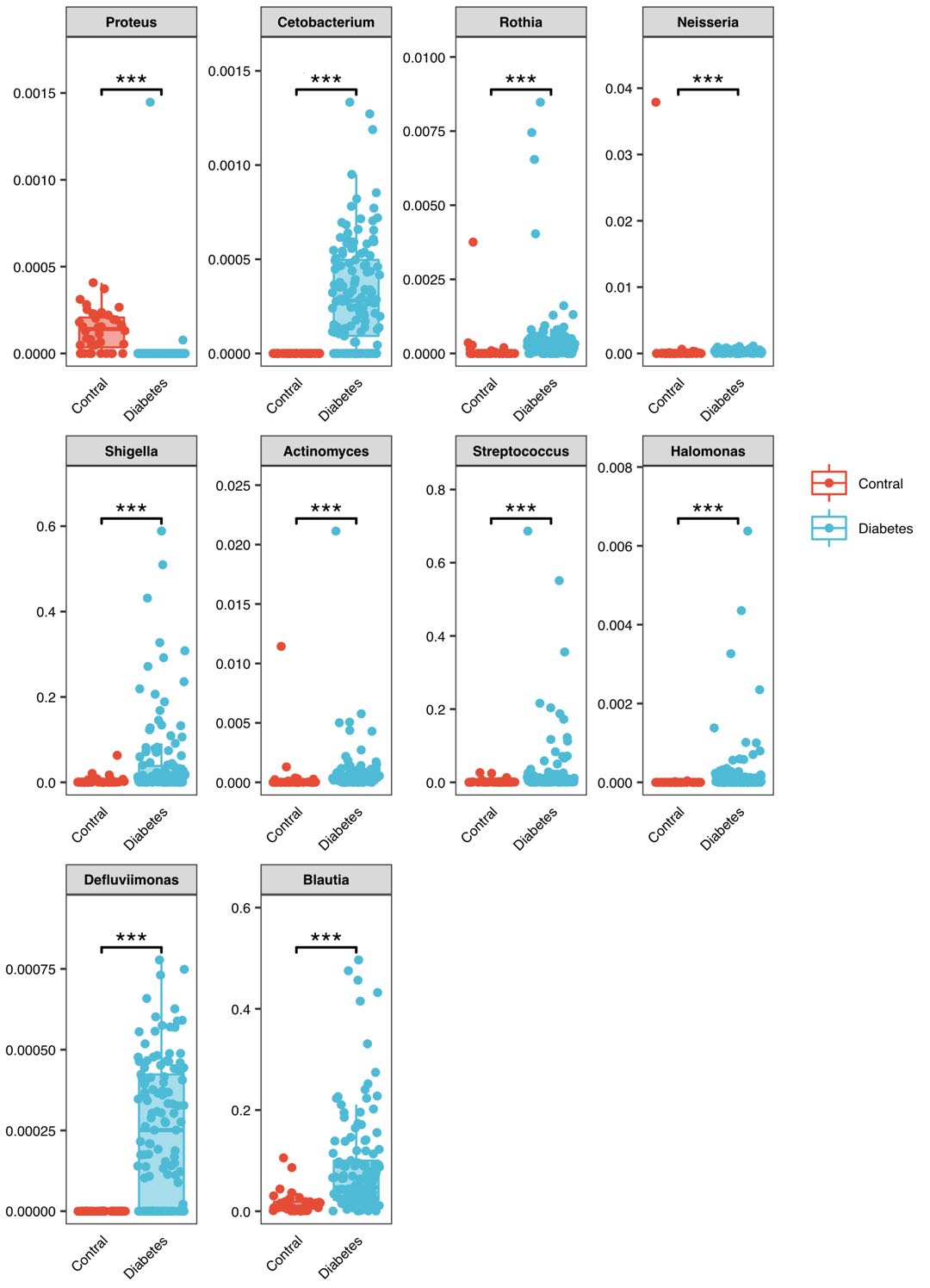


**Supplementary Figure 6. The difference of intestinal microbiota abundance between two groups** (***means *P* < 0.001)


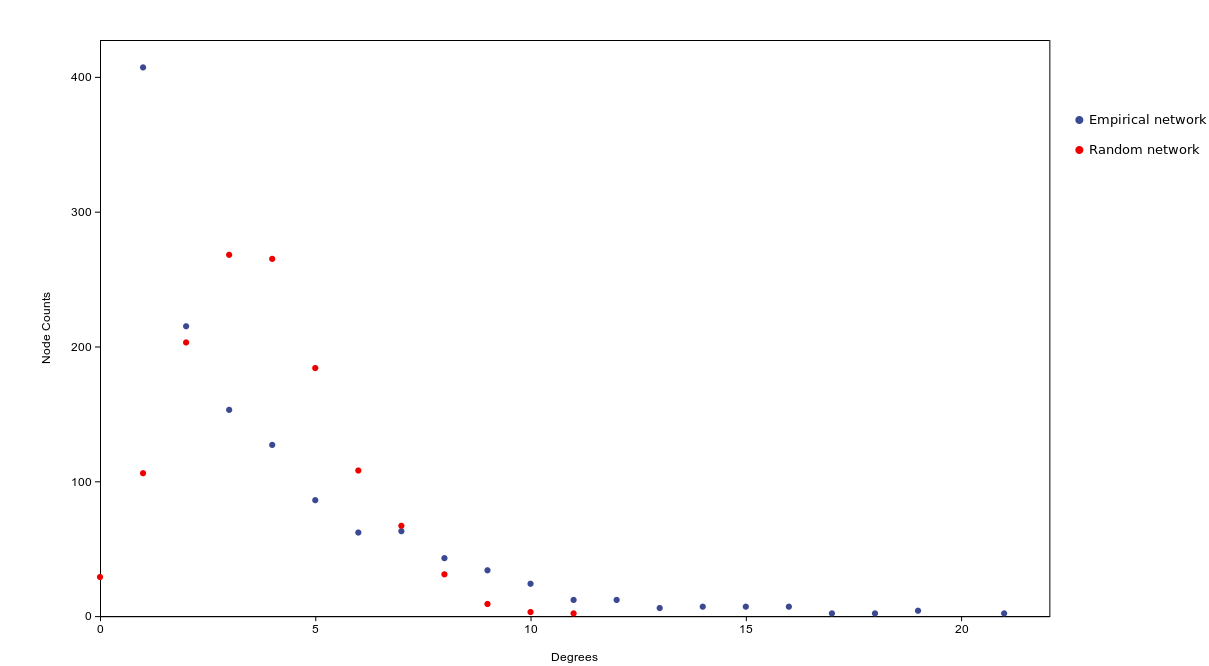


**Supplementary Figure 7. Degree distribution of intestinal flora in patients with type 2 diabetes（**The abscissa is the degree of the node, and the ordinate is the number of nodes with corresponding degrees.）

**
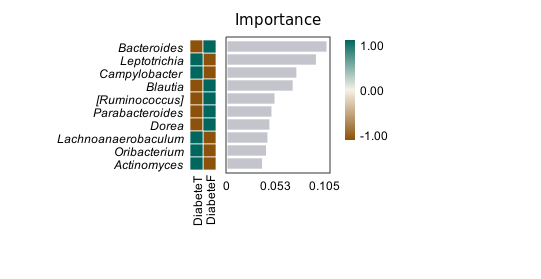
**

**Supplementary Figure 8. Random forest graph of oral and intestinal microbiota in T2DM**


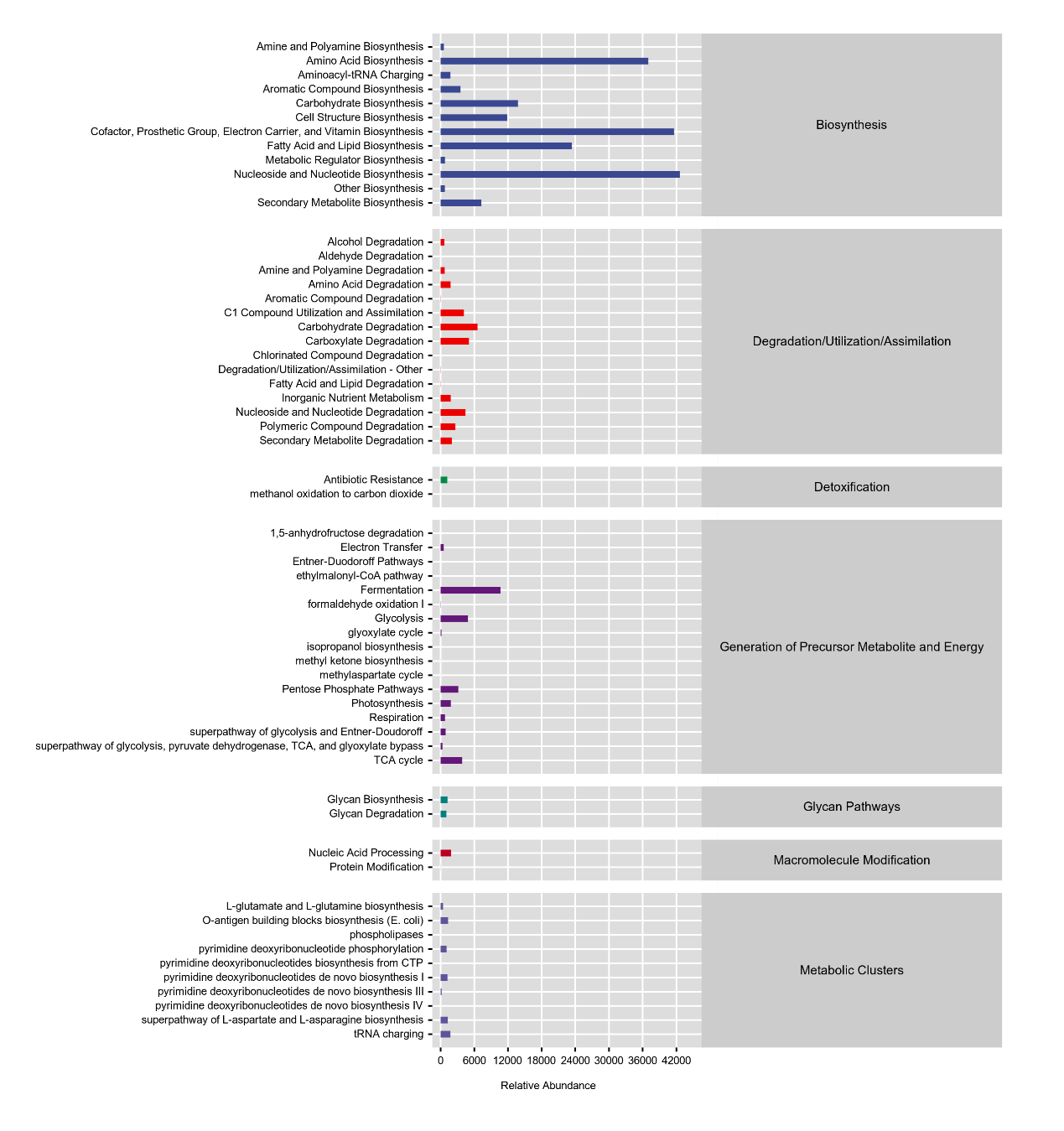


**Supplementary Figure 9. Prediction of metabolic pathways of tongue coating flora in patients with T2DM** (The abscissa is the average relative abundance of functional pathways, the ordinate is the functional pathway of MetaCyc at the second classification level, and the right side is the first level pathway to which the pathway belongs)


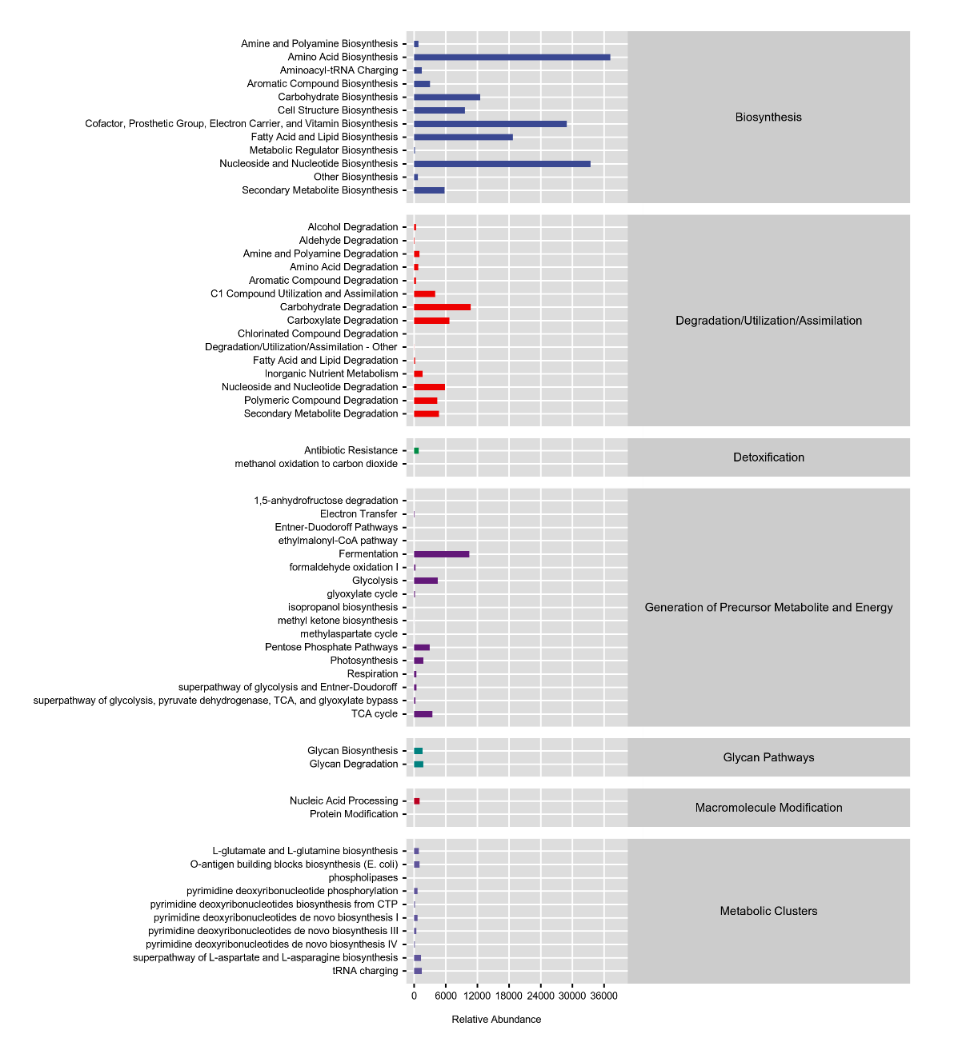


**Supplementary Figure 10. Prediction of metabolic pathways of intestinal flora in patients with T2DM**


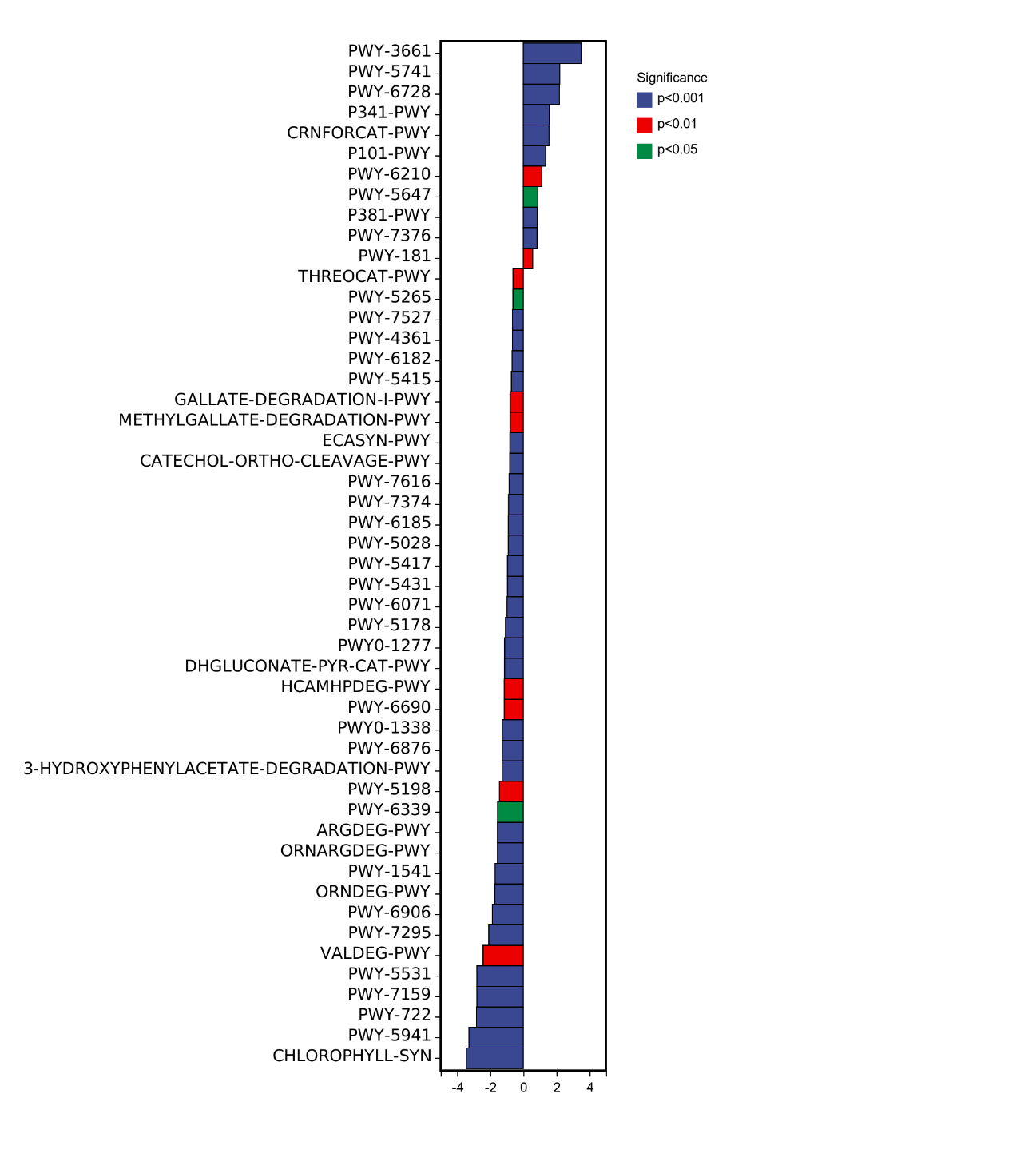


**Supplementary Figure 11. The distribution of significantly up-regulated and down-regulated metabolic pathways of the tongue coating microbiota in patients with T2DM**(The positive values are significantly up-regulated pathways, negative values are significantly down-regulated pathways, compared with the controls.)


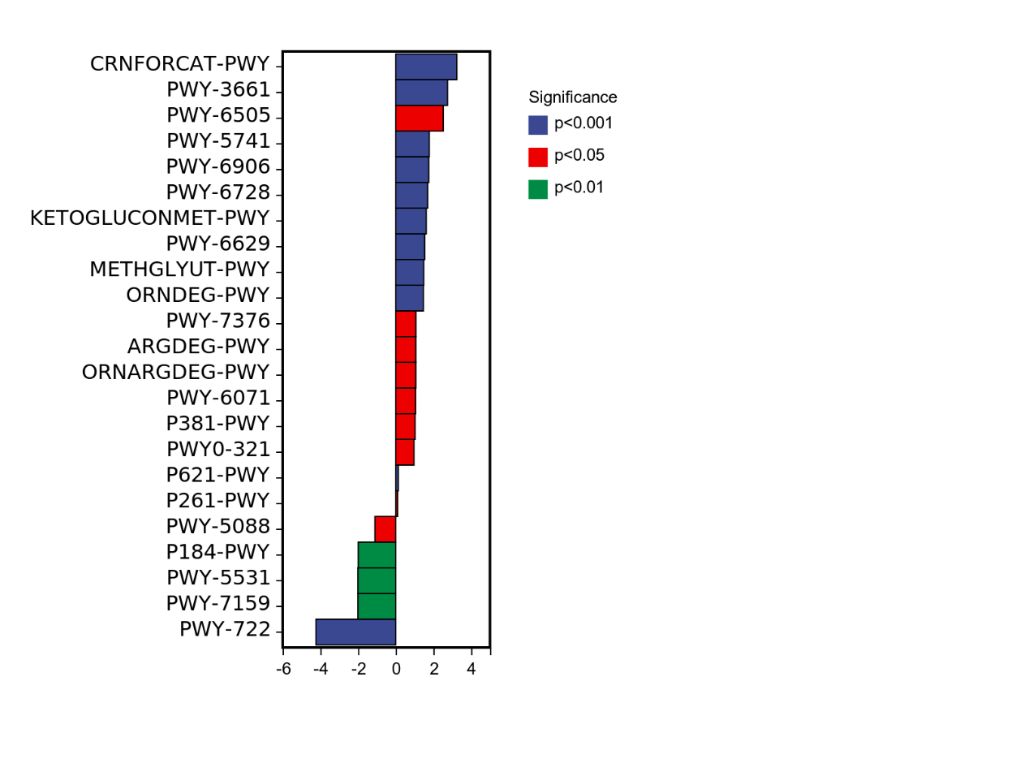


**Supplementary Figure 12. The distribution of significantly up-regulated and down-regulated metabolic pathways of the intestinal microbiota in patients with T2DM**

## Supplementary Tables

| **Supplementary Table 1** Basic information of diabetes group and control group [n (%) / M (P_25_, P_75_)] | | | | | |
| --- | --- | --- | --- | --- | --- |
| Groups | *n* | Gender | | Age | BMI |
|  |  | Male (%) | Female (%) |  |  |
| Diabetes | 183 | 108(59.0) | 75(41.0) | 59.6(48.2,68.0) | 25.0(22.6,27.8) |
| Controls | 74 | 26(35.1) | 48(64.9) | 25.5(20.0,50.3) | 21.6(19.6,24.0) |
| Note: This table provides information on all volunteers involved in this study | | | | | |

| **Supplementary Table 2** Microbiota structure differentiation between diabetes and controls  (The result of the PERMANOVA test) | | | | | | |
| --- | --- | --- | --- | --- | --- | --- |
| Group1 | Group2 | Sample size | Permutations | F | p-value | q-value |
| all | - | 257 | 999 | 10.78861 | 0.001 | - |
| ControlT | DiabeteT | 257 | 999 | 10.78861 | 0.001 | 0.001 |
| Note: T represents the tongue-coating microbiota | | | | | | |

| **Supplementary Table 3** Basic information of diabetes group and control group [n (%) / M (P_25_, P_75_)] | | | | | |
| --- | --- | --- | --- | --- | --- |
| Groups | *n* | Gender | | Age | BMI |
|  |  | Male(%) | Female(%) |  |  |
| Diabetes | 128 | 75(58.6) | 53(41.4) | 62.4(60.0,68.5) | 24.3(22.3,27.3) |
| Controls | 40 | 9(22.5) | 31(77.5) | 20.0(19.0,23.8) | 21.0(19.0,21.9) |
| Note: This table provides information on volunteers who had fecal microorganism detection in this study | | | | | |

| **Supplementary Table 4** Microbiota structure differentiation between diabetes and controls  (The result of the PERMANOVA test) | | | | | | |
| --- | --- | --- | --- | --- | --- | --- |
| Group1 | Group2 | Sample size | Permutations | F | p-value | q-value |
| all | - | 168 | 999 | 4.725636 | 0.001 | - |
| ControlF | DiabeteF | 168 | 999 | 4.725636 | 0.001 | 0.001 |
| Note: F represents the gut microbiota | | | | | | |

| **Supplementary Table 5** Microbiota structure differentiation between oral and gut  (The result of the PERMANOVA test) | | | | | | |
| --- | --- | --- | --- | --- | --- | --- |
| Group1 | Group2 | Sample size | Permutations | F | p-value | q-value |
| all | - | 311 | 999 | 61.93305 | 0.001 | - |
| DiabeteT | DiabeteF | 311 | 999 | 61.93305 | 0.001 | 0.001 |
| Note: T represents the tongue-coating microbiota; F represents the gut microbiota | | | | | | |

| **Supplementary Table 6** The correlation results of bacteria distributed both in the oral and intestine  (the top 10 phylum) | | |
| --- | --- | --- |
| Oral bacteria | Intestinal bacteria | r |
| Spirochaetes | Proteobacteria | 0.240** |
| SR1 | Spirochaetes | 0.195* |
| Verrucomicrobia | Verrucomicrobia | 0.222* |
| Spirochaetes | Spirochaetes | 0.180* |
| Note：This table only presents the results with statistically significance, *represents P≤0.05；** represents P≤0.01. | | |
